# Health literacy skills and the benefits of cardiovascular disease prevention

**DOI:** 10.1007/s12471-017-1001-4

**Published:** 2017-05-17

**Authors:** R. J. G. Peters

**Affiliations:** Department of Cardiology, Academic Medical Center, Amsterdam, The Netherlands

Cardiovascular disease (CVD) prevention can be highly effective: the complete elimination of health risk behaviours could potentially prevent around 80% of CVDs (and 40% of cancers) [1, 2]. Most of the effective treatments are relatively simple (although they may require extraordinary discipline), and most of the drugs that are prescribed are generic and highly affordable.

The greatest challenge is therefore in implementation of these preventive treatments. Consistently, surveys report low levels of adherence to lifestyle improvements and to medication, particularly in the long term. Even in secondary prevention, in patients who have experienced complications of CVD, adherence is disappointing [3]. Significant inequalities exist between countries and between ethnicities in the same country [4]. Among the many variables that impact on these patterns, socio-economic status is prominent.

In this issue, Van Schaik et al. report on one of the elements associated with low socioeconomic status: low health literacy [5]. According to the World Health Organisation, health literacy is linked to literacy and entails people’s knowledge, motivation and competences to access, understand, appraise and apply health information in order to make judgements and take decisions in everyday life concerning healthcare, disease prevention and health promotion to maintain or improve quality of life during the life course [6].

The authors reviewed data from the RESPONSE 1 study, which randomised patients who had been discharged after an acute coronary syndrome to a nurse-led secondary prevention program on top of usual care, or to usual care alone [7]. The overall finding of the RESPONSE 1 study was that the nurse-led prevention program significantly improved adherence to guideline-based preventive treatments, and reduced the (calculated) overall risk of clinical events. Nurse led care in secondary prevention is now recommended in European cardiology guidelines [8].

The current analysis shows that inadequate health literacy is highly prevalent in patients with coronary artery disease, ranging from 18% who have inadequate reading skills to 52% who have difficulty understanding and applying written information. Patients with low health literacy had significantly worse CVD risk profiles.

The findings are consistent with other reports [9, 10]. However, these studies were either performed in primary prevention or assessed single risk factors instead of integrated risk profiles. The investigation by Van Schaik et al. is the first to investigate the impact of health literacy on the effects of secondary prevention by nurse coordinated care. Importantly, patients with inadequate health literacy in the intervention group had improved risk profiles at 12-month follow-up, while those with inadequate health literacy in the control group showed no improvement. This suggests that the nurse-led prevention program was effective in patients with low health literacy, consistent with the overall results of the trial. Importantly, it remains to be established whether these effects persist in the longer term.

The current study did not address why the nurse intervention was effective. It is conceivable that particularly those who have a limited understanding of health information benefit from personal coaching by a nurse. In addition, it is unknown which approach is appropriate: education and information or behavioural modification, or combinations. More variables matter in this respect, including income, ethnicity, age, gender, all adding to the complexity of selecting and implementing the best approaches to prevention. Given the numbers of patients globally, and given the size of the potential impact of preventive treatments, these issues clearly deserve further study.
